# Muscone with Attenuation of Neuroinflammation and Oxidative Stress Exerts Antidepressant-Like Effect in Mouse Model of Chronic Restraint Stress

**DOI:** 10.1155/2022/3322535

**Published:** 2022-09-30

**Authors:** Hua Liu, Lian Lin Liu, Jing Chen, Yue Wen Chen, Yue Chai, Qing Shan Liu, Yong Cheng

**Affiliations:** ^1^Key Laboratory of Ethnomedicine for Ministry of Education, Center for Translational Neuroscience, School of Pharmacy, Minzu University of China, Beijing, China; ^2^Chinese Academy of Sciences Key Laboratory of Brain Connectome and Manipulation, Shenzhen Key Laboratory of Translational Research for Brain Diseases, The Brain Cognition and Brain Disease Institute, Shenzhen Institute of Advanced Technology, Chinese Academy of Sciences, Shenzhen-Hong Kong Institute of Brain Science, Shenzhen Fundamental Research Institutions, Shenzhen 518055, China; ^3^Shenzhen College of Advanced Technology, University of Chinese Academy of Sciences, Beijing 100049, China

## Abstract

Major depressive disorder (MDD) is a common mental disorder with high morbidity. Stress negatively affects for MDD development, whereby transport of stress-induced inflammatory mediators to the central nervous system (CNS) is associated with the etiology of mood disorders. Muscone is a pharmacologically active ingredient isolated from musk, with anti-inflammatory and neuroprotective effects. We hypothesized that muscone may ameliorate depression-like behavior by regulating inflammatory responses. To test this hypothesis, we used the chronic restraint stress (CRS) depression model, and CRS mice were treated with muscone (10 mg/kg, i.g., respectively) for 14 days. The effects of the drug on depressive-like behaviors were evaluated via the open field test (OFT), novelty-suppressed feeding test (NSFT), tail suspension test (TST), and forced swimming test (FST). Quantitative reverse transcription-PCR (qRT-PCR) was utilized to assess levels of proinflammatory cytokines (IL-6, TNF-*α*, COX2, and IL-1) and the anti-inflammatory cytokines (IL-4 and IL-10). We also determined levels of oxidative stress factors (malondialdehyde, superoxide dismutase, and glutathione peroxidase), as well as doublecortin (DCX) expression by immunofluorescence. The results showed that depression-like behavior and inflammatory levels were improved after muscone treatment. Muscone also significantly improved neurogenesis in the CRS mouse hippocampus and decreased oxidative stress in both the central and peripheral nervous systems. In conclusion, this work is the first to demonstrate that muscone has an antidepressant effect using a CRS model. Oxidative stress, neurogenesis, and inflammatory pathways are key factors affected by the drug and may represent new therapeutic targets to treat MDD, in this impact. These results may represent a new therapeutic target for MDD.

## 1. Introduction

The major depressive disorder (MDD) is a prevalent neuropsychiatric disorder, which is characterized by depressed moods or a sense of melancholy as well as a loss of interest or enjoyment in daily activities [1, 2]. Compared to other psychiatric disorders, the high prevalence of MDD poses widespread social concern. MDD significantly impacts patients' quality of life, and MDD is also the main contributor to psychiatric disability globally [3]. According to the World Health Organization (WHO) [4], depression rose to become the second most common cause of abnormal mortality and disability in 2020. Diseases associated with depression also create a heavy financial burden on MDD patients' family members and society [5–7]. Hence, the search for an effective treatment for depression is urgent and necessary.

Recently, accumulated evidence suggests that the occurrence of MDD disease is strongly elated to neurogenesis deficiencies, inflammatory responses, and oxidative stress [8–13]. NLRP3 (NOD-, LRR-, and pyrin domain-containing protein 3) inflammasome is a multimeric protein complex and an important component of the innate immune system as well as a crucial driver of neuroinflammation. The NLRP3 inflammasome is an intracellular sensor that detects damaging stimuli and activates caspase-1, which releases proinflammatory cytokines such IL-1*β* [14]. More importantly, NLRP3 inflammasome is strongly associated with neuroinflammation in depressive disorders [15]. Oxidative stress is a state of imbalance state between oxidation and antioxidation, which can lead to inflammatory infiltration and significantly increase the production of oxidative intermediates. Free radicals, reactive oxygen and nitrogen species, and oxidative stress have all been reported to play key roles in the pathogenesis of major depression [16]. The neurotrophic protein known as brain-derived neurotrophic factor (BDNF) is highly expressed throughout the nervous system. Over the last decade, several studies have consistently suggested that BDNF is strongly linked to antidepressants [17]. Recent studies demonstrated that microglia are closely associated with depression development and BDNF can target microglia to show antidepressant effect [18, 19]. In addition, BDNF can correct NLRP3 inflammasome through the KLF2/HK1 pathway [20]. Taken together, BDNF activation of the NLRP3/caspase-1 pathway may contribute to the treatment of MDD patients.

Musk from musk deer is one of the most precious traditional Chinese medicinal resources and has been used to treat neuropsychiatric diseases for many years [21, 22] Muscone is a key active ingredients in the musk and is known to be neuroprotective. However, the use of muscone to treat MDD is limited, and the mechanism by which muscone can inhibit NLRP3 inflammasome activity has yet to be resolved. In this study, the antidepressant-like properties of muscone were examined using a mouse model of chronic restraint stress (CRS) to represent MDD.

## 2. Material and Methods

### 2.1. Animals

Forty-seven-week-old healthy C57BL/6 male mice (18-22 g) were provided by Fangyuanyuan Breeding Farm (Beijing, China). The mice were kept in a cage at a constant temperature of 23 ± 2°C, with a 12 h light/12 h light-dark cycle. Over the week preceding the start of the drug trial, all mice had free access to food, water, and adaptive feeding. All experimental protocols adhered to the Guidelines of the Animal Care and Use Committee at Minzu University of China (ECMUC2019001AO).

### 2.2. The Study Design

**Figure 1:**
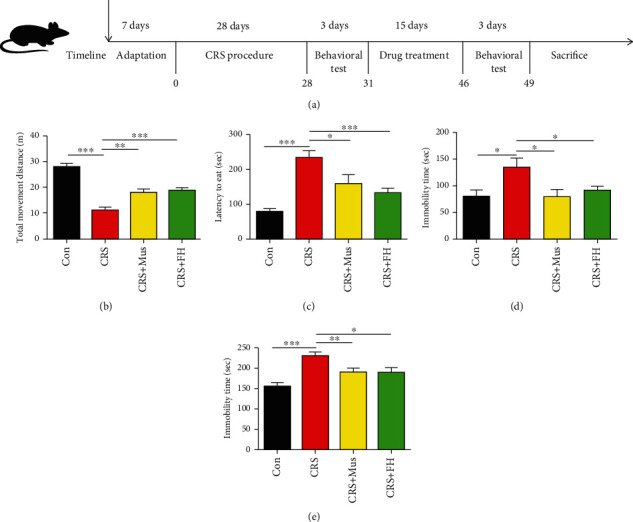
In the CRS-induced mouse model, muscone reduced depressive-related behaviors. (a) Experimental program for the model of depression caused by the CRS. (b) Muscone restored the depressive-like behaviors in OFT. (c) Muscone restored the depressive-like behaviors in the NSFT. (d) Muscone-treated mice exhibit less depression-like behavior in TST. (e) Muscone-treated mice exerted less depression-like behavior in the FST. Data are presented as mean ± SEM, with 10 mice in each group. ^∗^*p* < 0.05, ^∗∗^*p* < 0.01, and ^∗∗∗^*p* < 0.001 compared to the CRS group.

We chose to use the CRS depression model previously described but with modifications [23]. Briefly, the CRS group mice were sedated for 8 hours each day for 28 days and were restrained in cylindrical tubes. At the end of the restraining period, the mice were immediately assigned to cages with free access to food and water. The CRS model mice were subsequently divided randomly into three groups at random: a control group (CRS, *n* = 10); a group that received intragastrically administered muscone (CRS+MUS, *n* = 10; 10 mgkg^−1^24 h^−1^); and a group that received fluoxetine (CRS+FH, *n* = 10; 10 mgkg^−1^24 h^−1^). During administration of muscone, the CRS group, the CRS+MUS group, and the CRS+FH group were restrained in tubes for 2 h to maintain the depression-like behavior. The control group (Con, *n* = 10) mice received normal saline during administration without any treatment. After 15 days of drug therapy, the mice underwent a behavioral test before being euthanized. Blood serum and brain tissues were stored at -80°C for subsequent biochemical tests. The animal experiment flow chart is shown in Figure 1(a).

### 2.3. Behavioral Assessment

#### 2.3.1. Open Field Test (OFT)

The OFT is a well-known test for assessing depressive-like behavior as well as independent behavior in unfamiliar settings. The test was carried out in an open field apparatus (50 × 50 × 45 cm). The open field device has a black plate surrounding it and contains nine equal white square grids. Each mouse was put into a box corner and watched for 6 minutes under a fluorescent light. An open field system (Chengdu Taimeng Software Co. Ltd., Sichuan) measured and evaluated the total distance traveled over a period of five minutes.

#### 2.3.2. Novelty-Suppressed Feeding Test (NSFT)

The mice were starved for 24 hours before being subjected to the test. White filter paper was placed in the middle of the testing device (50 × 50 × 45 cm), and food particles were placed on the paper. Mice were positioned individually at the corner grid of the testing device floor and given five minutes to roam at will. The period of inactivity when the mice started to eat was recorded.

#### 2.3.3. Tail Suspension Test (TST)

The TST is one strategy used to test the learned helplessness phenomenon in MDD model mouse. The mice were separated, and each was strung with a white rope for the test. The mouse's head was 15 cm from the floor, and the rope was positioned 1 cm from the tail's tip. The last five minutes of the mouse's immobility was timed, while it was suspended for a total of six minutes. When a group of mice completed the test, the system was cleaned with 75% alcohol before the next mouse was positioned to take the test.

#### 2.3.4. Force Swimming Test (FST)

The forced swim test was conducted as previously described [7]. Each mouse was briefly placed in a cylindrical container with a diameter and height of 15 cm and a water temperature of 23 ± 2°C. All mice were forced to swim for 6 minutes, and the time spent immobilized at different time points following the 6-minute swim time was noted.

### 2.4. Quantitative Real-Time PCR (qRT-PCR)

Total RNA was extracted from the hippocampus using an mRNA isolation kit from Beijing Zhuangmeng International Biological Gene Technology Co., Ltd.; total RNAs were extracted from the hippocampus and then reverse transcribed into cDNA using Prime ScriptTM RT Master Mix. qRT-PCR was performed on a LightCycler® 96 system to detect the expression levels of selected proinflammatory and anti-inflammatory genes. The selected genes and gene-specific primer sequences are shown in Table 1. The cycling conditions consisted of 120 s at 95°s, followed by 40 cycles of 15 s at 95°C, 20s at 60°C, and 40s at 72°C, and melting conditions of 10s at 95°C, 60s at 65°C, and 1 s at 97°C.

### 2.5. Western Blotting

RIPA buffer containing a protease inhibitor cocktail was used to extract the total protein, and a bicinchoninic acid (BCA) protein quantification kit was used to calculate the protein concentration. Each sample's 40 *μ*g of protein was separated on an SDS-PAGE gel and then transferred onto nitrocellulose filter membranes. The membranes were incubated with the primary antibody overnight in a freezer at 4°C, following blocking solution incubation. The membranes were incubated with the HRP-conjugated secondary antibody for 1 hour at room temperature. The membranes were subjected to three 10-minute washes with TBST (Beijing Yuanping HAO Biotechnology Co., Ltd.) and then scanned with a chemiluminescence imager analysis system. The ImageJ software was used to analyze the individual protein bands.

### 2.6. Detection of Oxidation-Associated Markers

Oxidation-associated markers were determined using enzymatic colorimetric following the manufacturer's instructions. Briefly, mice's retroorbital vessels were used to collect peripheral serum samples (0.8 ml), which were then used to get serum layers (300 *μ*l) by centrifuging them for 20 minutes at 4°C at a speed of 4000 rpm. To determine the level of oxidative stress, samples of blood serum and hippocampus tissue samples were obtained from the mice. Using a kit from the Nanjing Jiancheng Bioengineering Institute, we assessed the amounts of malondialdehyde (MDA), glutathione peroxidase (GSH-Px), and superoxide dismutase (SOD).

### 2.7. Immunofluorescence

Animals were sacrificed by intracardial perfusion with saline, and mice brains were fixed with 4% paraformaldehyde. The fixed brains were dehydrated once in 20% sucrose and twice in 30 percent sucrose (both in PBS). The coronal sections were cut to a thickness of 35 *μ*m, and the sections were washed with 1x PBS. The sections were blocked with blocking buffer (1% BSA+0.3% Triton X-100+10% goat serum in PBS) for one hour at room temperature after three washes in 1x PBS. The coronal sections were then incubated with the primary antibody anti-DCX: (1 : 400, Cell Signaling Technology, #14082) for an additional overnight period at 4°C, followed by an additional two hours at room temperature with the fluoroconjugated secondary antibodies. Sections were washed three times and stained with DAPI (4′,6-diamidino-2-phenylindole) solution. Finally, images were captured with a Leica TCS SP8 confocal microscope (Leica Microsystems, Germany).

### 2.8. Data Analysis

Software called GraphPad Prism 5.0 was used to conduct statistical analysis. One-way analysis of variance (ANOVA) and unpaired independent sample tests were used to determine the significance between groups. *p* < 0.05 was regarded as statistically significant, and all data are presented as means ± SEM.

## 3. Results

### 3.1. Effect of Muscone Treatment on Behavior Performance

The OFT test was used in this investigation to examine how muscone therapy affected CRS-induced depressive-like behavior that in mice. The outcome shows that the mice in the CRS group traveled shorter distances than the animals in the Con group. When compared to the CRS group, mice treated with muscone did exhibit a significantly longer travel distances (*p* < 0.01, Figure 1(b)). Furthermore, In the NSFT assessment, as respected, muscone restored the time spent to search for food (*p* < 0.05, Figure 1(c)). The TST and FST were carried out to explore the desperate behavior of the tested mice. According to Figures 1(d) and 1(e), mice with CRS significantly reduced their immobility durations during the TST and FST tests. However, the surprising thing is that the muscone treatment can significantly restore the behavior change of MDD mice (*p* < 0.05, Figure 1(d), and *p* < 0.01, Figure 1(e)).

### 3.2. Muscone Ameliorates CRS-Induced Oxidative Stress In Vivo

**Figure 2:**
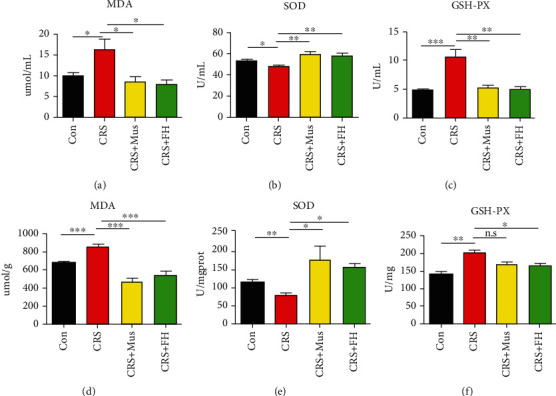
shows how the muscone therapy affected the antioxidant capacity of mouse serum and hippocampal tissues. (a–c) The kit was used to measure the enzymatic activity or levels of serum SOD, MDA, and GSH-Px. (d–f) The kit was used to assess the SOD, MDA, and GSH-Px enzymatic activity or levels in the hippocampus. Data are presented as mean ± SEM, with 7 mice in each group. n.s: not significant; ^∗^*p* < 0.05, ^∗∗^*p* < 0.01, and ^∗∗∗^*p* < 0.001 compared to model group.

MDA levels are frequently utilized as a measure of harm caused by oxidative stress. According to this study's findings, mice in the CRS group had significantly higher levels of MDA in their peripheral serum and hippocampus (*p* < 0.05 or 0.001). While in mice treated with muscone (10 mg/kg) and FH, the MDA contents showed significantly decreased changes in both peripheral serum and the hippocampus (*p* < 0.05 or 0.001, Figures 2(a) and 2(d)). SOD content is a vital component of antioxidant enzyme system in biological system. According to the study's findings, mice in the CRS group had significantly higher levels of MDA in their peripheral serum and hippocampus (*p* < 0.05 or <0.01). Like FH, muscone can significantly increase the SOD levels in both the peripheral serum and the hippocampus (*p* < 0.01 or <0.05, Figures 2(b) and 2(e)). Another significant peroxide found throughout the body is GSH-Px. It can convert harmful peroxides into harmless hydroxyl molecules and catalyze the conversion of GSH into GSSG. H_2_O_2_ can be broken down more quickly with the help of GSH-PX, and the cell membrane is shielded from oxidant interference and harm. Our results demonstrated that muscone was effective in increasing GSH-Px levels (*p* < 0.01 or >0.05, Figures 2(c) and 2(f)). The analysis of the data is described in Table 2.

### 3.3. Effect of Muscone on Inflammatory Cytokines

**Figure 3:**
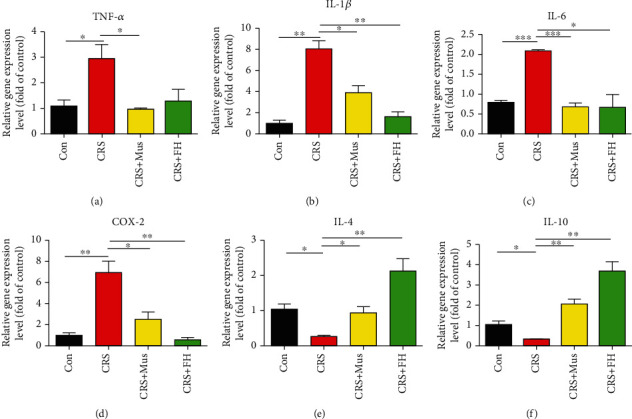
Effect of muscone on anti-inflammatory proteins in the hippocampus. TNF-*α*, IL-1*β*, Il-6, IL-10, COX2, and IL-4 levels were identified by qRT-PCR in the hippocampus tissue. Values are expressed as mean ± SEM, with 3 mice in each group. n.s: not significant; ^∗^*p* < 0.05, ^∗∗^*p* < 0.01, and ^∗∗∗^*p* < 0.001 compared to the CRS group.

Compared to the control group, proinflammatory cytokines (IL-1*β*, IL-6, COX2, and TNF-*α*) were significantly increased, and anti-inflammatory factors (IL-10 and IL-4) were significantly decreased in the CRS group. Muscone treatment significantly reduced inflammatory cytokine levels in the CRS+Mus group as compared to the CRS group (Figures 3(a)–3(f)).

### 3.4. Effect of Muscone Treatment on the NLRP3 Pathway

**Figure 4:**
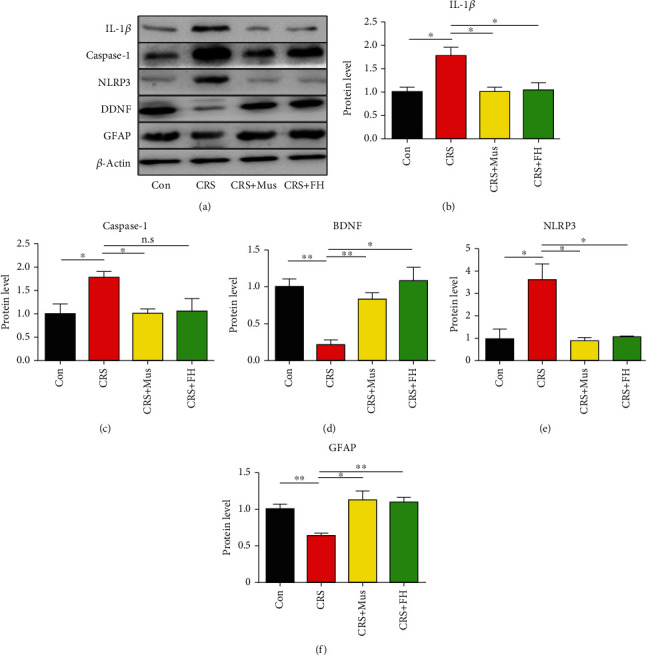
(a) Muscone attenuates CRS-induced NLRP3 signaling ways in mice. (b and c) BDNF and GFAP protein levels as measured from the western blots. (d–e) Protein levels in the NLRP3/caspase-1 signaling axis were detected using western blot. Values are expressed as mean ± SEM, with samples from 3 mice in each group. n.s: not significant; ^∗^*p* < 0.05, ^∗∗^*p* < 0.01, and ^∗∗∗^*p* < 0.001 compared to the CRS group.

Compared with the control group, CRS induced significant overregulated protein expression of NLRP3 in the model group. The expression of the NLRP3 protein was considerably lower in the CRS+MUS group, in contrast to the model group (Figure 4). Muscone treatment can significantly reduce IL-1*β* protein expression levels induced by CRS. Caspase-1 in the hippocampal region of the muscone-treated group was predominantly downregulated compared to the CRS group (Figure 4).

### 3.5. Effect of Muscone Treatment on Neurogenesis

**Figure 5:**
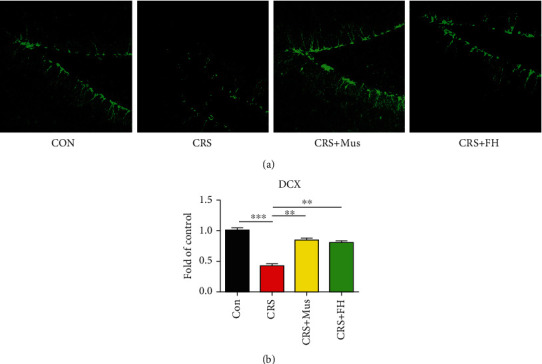
Effect of muscone treatment on DCX expression in the hippocampus. (a) Confocal photomicrographs of DCX-immunostained immature neurons. (b) Quantification of DCX-positive cells. Data are presented as mean ± SEM, *n* = 3. ^∗^*p* < 0.05 and ^∗∗^*p* < 0.01 compared to model group.

Next, we further assessed how muscone affected hippocampus neurogenesis using an immunofluorescence assay to gauge expression of DCX, a surrogate marker of neurogenesis analysis (Figure 5(a)). We discovered that in the CRS group of mice, DCX expression in the hippocampus was significantly reduced and that the reduction was reversed following muscone administration (*p* < 0.01, Figure 5(b)). It indicated that the neuroprotective effect of muscone is related to its increasing neurogenesis.

## 4. Discussion

Traditional Chinese medicine played a crucial role in drug research and development and clinical treatment of antidepressants with the characteristics of less adverse reactions, multiple effects, and multiple targets. There is an actual significance to study the antidepressant effect and its mechanism of action of traditional Chinese medicine. Muscone has been shown to treat neurological disorders and chronic inflammation; however, its ability to treat role on depression remains to be explored. In the current work, we discovered that the multitarget medication muscone can diminish the behavior patterns associated with depression in a CRS-induced mouse model. Through further molecular experiments, we found that the underlying potential mechanism of muscone as an antidepressant was associated with its anti-inflammatory, antioxidation, and nerve regeneration properties. This also indirectly suggests that muscone is a tentative option for future research into alternative treatments for depression.

Clinical and preclinical evidence suggested that stress was closely associated with the development of depression [24]. Stress is known to induce release of proinflammatory cytokines, a hallmark of depression in an animal model [25]. To confirm the therapeutic benefits of muscone on CRS-induced depression, we adopted this model of chronic restraint stress for this investigation. After 28 days of being shackled, the mice showed depression-like behavior. More importantly, the pathological changes in CRS model mice were observed that acute or chronic inflammation spread throughout hippocampus areas and oxidative stress factors similarly activated. These changes are consistent with the CRS-induced depression model features [7] .

To explore the potential antidepressant mechanism of muscone, we focused on the effects of muscone on neuroinflammation and loss of neurons. Results from the mouse model showed significant improvement in motor function and cognitive deficits in the mice with depression after muscone treatment. Moreover, inflammatory factors that were notably increased in the hippocampus areas were decreased after muscone treatment. The findings imply that muscone can prevent the hippocampus from producing more proinflammatory cytokines. In addition, we also explored the effect of the muscone on neuronal regeneration. We found that the number of newborn neurons increased significantly after treatment with muscone. Thus, the role of muscone in antidepressant is associated with neurological function recovery in a mice model of MDD. Further attenuation of the inflammatory responses promoted neurogenesis in the hippocampus area.

Stress has been shown to induce neuroinflammation. In neuroinflammation, the NLRP3 inflammasome has an important responsibility for the number of innate immune processes associated with infection neuroinflammation and autoimmunity. In addition, NLRP3 inflammasome signaling leads to an inflammatory response in the central nervous system (CNS), which can harm neuronal cells [26]. Hence, in this study, we focused on the NLRP3 pathway. The NLRP3 inflammasome is the most thoroughly studied inflammasome complexes that have been described up to now [27]. There is evidence to support the idea that NLRP3 inflammation is important in the pathophysiology of major depression [28] IL-1*β* is a key inflammatory cytokine that can promote a variety of autoimmune inflammatory reactions and cell activities, including cell proliferation, differentiation, and apoptosis [29]. NLRP3 activation can promote the activation of caspase-1 and triggered IL-1*β* production [30, 31]. One study found that antidepressant compounds decrease NLRP3 activation [32]. To further investigate the underlying mechanism, the classical inflammatory pathway, NLRP3 inflammation signaling pathway, attracted to our attention. Our data support the previous conclusion. Our outcomes showed that the expression level of NLRP3 protein, caspase-1 protein, and IL-1*β* protein decreased in varying degrees after muscone treatment. In conclusion, this also indirectly indicates that the antidepression effect of muscone acts via downregulating the expression of the NLRP3 protein, caspase-1 protein expression, and IL-1*β* protein to activate the NLRP3 inflammation signaling pathway activation.

Oxidative stress is created by an imbalance between oxidation and antioxidation in the body, which consequently leads to oxidize tissue damage. A growing number of evidence suggest that increased oxidative stress plays a significant role in the etiology of MDD [33]. Based on our findings, the muscone group saw a significantly lower level of MDA and GSH-Px as well as a significantly higher level of SOD following muscone treatment. Those outcomes demonstrated that the level of oxidative stress was attenuated. Hence, this data firmly supports our earlier conclusion.

This study has some limitations. Firstly, a previous study has shown that 10 mg/kg of muscone has neuroprotective roles. We elected to use this single dose to explore the antidepression effect. Although the dose of muscone 10 mg/kg was an effective dose, we did not evaluate different doses to determine the optimal dose of muscone as an antidepressant. Thus, the optimum dose of muscone as an antidepressant requires further investigation. Secondly, although the CRS depression model CRS depression model is similar to the clinical characteristics of human MDD, the model shows a trend of self-recovery. For those reasons, we restrained the animals for two hours during the muscone administration phase. This experimental design can alleviate the problem of self-recovery to some extent but differs from the actual clinical appearance. In addition, we also examined neurogenesis in the hippocampus and certain indicators. However, the relationship between neurogenesis and those indicators is unclear. The potential mechanism affected by muscone requires further verification.

## 5. Conclusion

**Figure 6:**
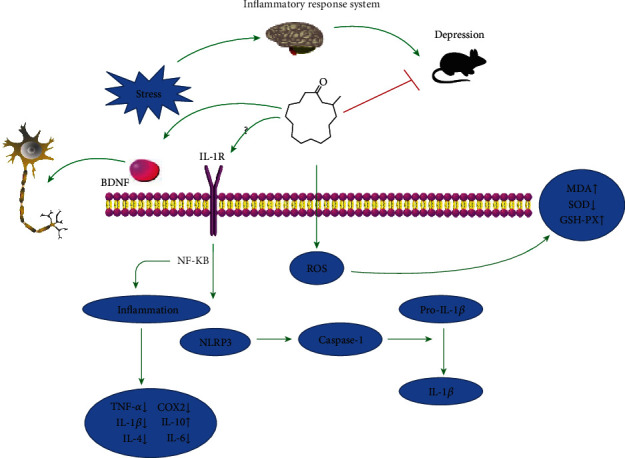
Potential mechanism of the muscone antidepressant effect.

In summary, the finding of our present study suggested that muscone treatment can alleviate CRS-induced depressive behavior. Numerous results demonstrate that the antidepressive characteristics of muscone are linked to its ability to reduce inflammation, minimize oxidative stress, and support the nerve regeneration signaling system (Figure 6). Muscone is a multitarget antidepressant that may be more successful than medications with a single target drug. This conclusion provided further insights into the pathogenesis of antidepression of muscone and indicates that muscone is a kind of potential antidepressant drug.

## Figures and Tables

**Table 1:** Gene-specific primers for qRT-PCR.

Genes	Primers (5′-3′)	
TNF-*α*	Forward	GGCTTTCCGAATTCACTGGAG
	Reverse	CCCCGGCCTTCCAAATAAA
IL-1*β*	Forward	AGCTGGAGAGTGTGGATCCC
	Reverse	CCTGTCTTGGCCGAGGACTA
IL-6	Forward	CACAGAGGATACCACTCCCA
	Reverse	GAATTGCCATTGCACAACTCT
COX2	Forward	GCCAGCAAAGCCTAGAGCAA
	Reverse	GCCTTCTGCAGTCCAGGTTC
IL-4	Forward	AACGAAGAACACCACAGAGAG
	Reverse	ATCGAAAAGCCCGAAAGAGT
IL-10	Forward	GGCGCTGTCATCGATTTCT
	Reverse	GCCTTGTAGACACCTTGGTC
*β*-Actin	Forward	AAGCCCTGGATGAAGAAACAG
	Reverse	TGGGAACCAATCTCGTAGGTC

**Table 2:** The effect of muscone treatment on antioxidant ability in serum and hippocampus tissues of mice.

Item	Sample	Con	CRS	CRS+Mus	CRS+FH
MDA	Serum (U/ml)	9.85 ± 2.06	16.26 ± 6.30	8.43 ± 3.26	7.93 ± 2.58
	Hip (U/mg)	676.83 ± 52.88	853.68 ± 72.55	464.89 ± 109.29	537.175 ± 128.15
SOD	Serum (U/ml)	53.26 ± 3.71	48.01 ± 3.22	59.38 ± 7.06	58.00 ± 5.47
	Hip (U/mg)	115.93 ± 18.95	79.05 ± 16.06	177.66 ± 16.06	157.43 ± 61.20
GSH-PX	Serum (U/ml)	4.81 ± 0.45	10.64 ± 3.08	5.19 ± 1.52	4.92 ± 1.14
	Hip (U/mg)	140.95 ± 13.51	198.71 ± 30.68	168.33 ± 17.20	164.29 ± 21.42

## Data Availability

All data are contained within the manuscript.
